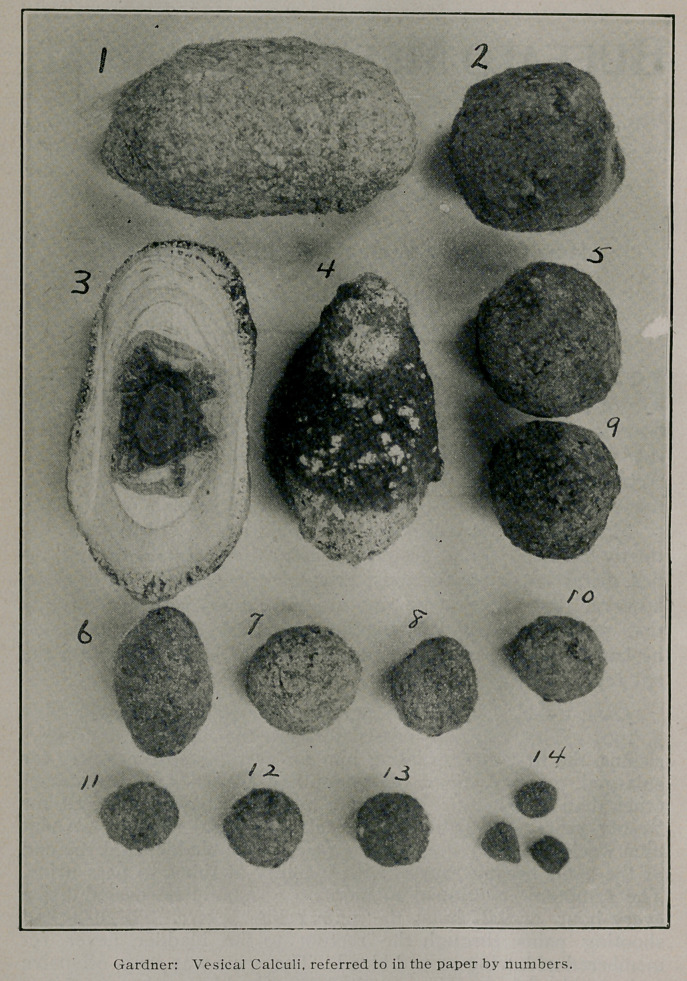# How Often Are Vesical Calculi Overlooked?1Read at the meeting of the Section on Surgery of the Buffalo Academy of Medicine, May 7, 1907.

**Published:** 1907-08

**Authors:** James A. Gardner

**Affiliations:** Surgeon to Genitourinary Department, Emergency Hospital, Buffalo, N. Y.


### BUFFALO MEDICAL JOURNAL.

VOL. LXIII. AUGUST, 1907. No. 1

### ORIGINAL COMMUNICATIONS.

### How Often are Vesical Calculi Overlooked?[1]

**With report of ten cases.**

By JAMES A. GARDNER, M.D.
Surgeon to Genitourinary Department, Emergency Hospital, Buffalo, N. Y.

IT is not possible to state even approximately the frequency of the presence of stone in the bladder. Nor can it be stated how often the condition is overlooked, but there can be no question that the condition is not recognised in a great many cases. According to the textbooks the symptoms are so plain and well marked that one almost wonders that it is possible to overlook or be misled in the diagnosis of a condition so clearly and distinctly defined. Pain, frequent micturition, hematuria and reflex disturbances are the most prominent symptoms. It is clear, however, that these symptoms are indications rather of cystitis than stone. I believe it is Bigelow, when asked how it happened he had so many cases of stone, who should be credited with the apt reply "I look for them."

CASE I.—H. J.—German, age 66, referred by Dr. Gould, May 3, 1901. This man had passed through the hands of four physicians, the first three treating him for cystitis and the fourth for enlarged prostate, none of whom had passed any instrument other than a catheter. He gave a history of having suffered for twenty years with pain in the back, loins and bladder. About nine weeks before I saw him the pain in the bladder and the end of the penis became worse, with inability at times to pass urine. The symptoms continued to increase. Usually he passed urine every hour or half hour, the pain being severe. He also had shooting pains through the rectum to the bladder, never remembered of having passed blood. The urine was ropy, offensive and contained pus. He consulted Dr. Gould two days previous and complained of severe pain which was almost continuous.

1. Read at the meeting of the Section on Surgery of the Buffalo Academy of Medicine, May 7, 1907.

Examination showed that the patient was weak and feeble with enlarged prostate; four ounces of residual urine and marked

Gardner: Vesical Calculi, referred to in the paper by numbers.

cystitis. Upon passing a Thompson searcher a stone was discovered of moderate size. He was operated on May 7, 1901, by perineal incision, the neck of the bladder was well stretched by uterine dilator and a stone (No. 5) weighing 13 grms. was removed. The prostate was not removed at this time owing to the man's poor condition. A perineal tube was placed in the wound and his bladder washed every four hours for two days and three times a day thereafter. The tube was removed on the fourth day, a self retaining catheter placed in the urethra and patient placed in semi-reclining position. The bladder was then washed three times a day for a week and daily for the following three weeks. As his strength returned there was such an improvement in his bladder symptoms that the operation on the prostate was postponed indefinitely. Three months after the operation he was catheterising himself every four hours and felt so much better that he refused further operative procedure. He had gained ten pounds in weight and continued very comfortably until his death from grippe in 1905.

Case 2.—J. E.—German, upholsterer, age 22, referred by Dr. Dorr. Patient came to the Emergency Hospital complaining of pain in bladder, a sense of soreness through the penis, and a constant desire to urinate which was unrelieved by the few drops of urine expelled from time to time. He was pale, emaciated, shaky, his eyes sunken and his lips dry and cracked. According to his own story he had been to ten doctors during the previous five years. Before coming to the Emergency Hospital he had been to one of the hospitals in the city where his case had been diagnosed as stricture and sounds were passed.

Upon examination a Thompson searcher revealed a large stone. Patient was operated upon on February 26, 1902, through superpubic incision. The bladder walls were found very much thickened and the stone (No. 3) weighing 57 grms. which was covered with sharp spiculae, was removed with difficulty. The specimen shows beautifully the formation with its neucleus of uric acid and concentric layers of phosphates. The patient made uninterrupted recovery and gained thirty pounds during the three months following the operation.

Case 3.—H. R.—American, janitor, age 59. History showed necessity to get up every hour or two during the night to urinate and during the first couple of hours after arising in morning urinated every fifteen minutes, after which time he was able to hold urine about two hours throughout the day. This history extends over six months. Patient had enlarged prostate. Three ounces of residual urine were found in the bladder. An examination of the bladder was not made either with a cystoscope or searcher because the prostate apparently was the cause of all the trouble, with the result that the stones were not discovered until the operation. This tends to prove that too great care cannot be exercised in an examination.

Patient entered the Emergency Hospital July 8, 1903, and operation was performed the following morning. The prostate was removed by perineal incision. When I introduced my finger into the bladder to explore it I found one stone (No. 6) in the post prostatic pouch and a second (No. 13) encysted in the anterior wall of the bladder above the urethreal outlet which was easily removed. Perineal drainage was placed in the wound and the bladder washed three times daily for four days with boric acid solution when the tube was removed and the patient ordered out of bed. He returned home at the end of two weeks and has had no subsequent trouble.

Case 4.—L. F.—German-American, clerk, age 30. First saw this patient in September, 1902, in consultation with Dr. McLaurie. He had a small caliber stricture upon which I advised an operation. Did not see him again until June 7, 1904. He gave history of gonorrhea five years previous; was operated on for stricture shortly after I saw him in 1902; complained of frequent urination ever since he had gonorrhea and of pain referred along the under surface of penis during two years, which at times was severe and again scarcely noticeable. The urine had looked dirty ever since the operation but during the last winter while he was under treatment for cystitis had cleared up somewhat, but the cystitis had returned as soon as he ceased treatment.

At the time of consultation in 1904, he complained of pain along the under surface of the penis before, during and after the act of micturition. Examination showed stricture in the pendulant portion, which was dilated sufficiently to pass a No. 16 F sound, and also one in the deep urethra which was too sensitive to admit an instrument. He was sent to bed and given urotropin grains 5 four times a day. After three days he seemed much improved and got up, but on moving about his pain and frequent micturition returned. I was able to pass a searcher at this time and had no difficulty in getting the click.

A perineal section was made June 18, 1904. and two stones (Nos. 7 and 9) removed, bladder drained for five days, washed twice a day with boric acid solution, a self retaining catheter placed in the urethra and patient permitted to get out of bed. Patient washed his bladder twice a day for the following week, at which time the wound had closed and catheter was removed. I saw him frequently during the subsequent three months while he was having sounds passed. He had no cystitis and no pain along the under surface of the penis. Saw him again in December, 1906, and his trouble had not returned.

Case 5.—A.B.—American, age 16, consulted me October 6, 1904. Gave history of masturbation during the previous two years, frequent urination, loss of fifteen pounds during the previous year, pain over the bladder and had passed blood a few times after urination. Patient had consulted three physicians, all of whom told him that his frequent urination and passing of blood was caused by his masturbation. No instrumental examination had been made.

Examination showed small meatus and urethra and it was with difficulty that a searcher was passed. The click was heard and a large size stone diagnosticated. I did not see him for some months when he returned to my office willing to have anything done that would afford relief. Suprapubic cystotomy was performed on January 9, 1905, and found the bladder walls thickened and the mucus membrane was in soiled condition. A stone (No. 1) weighing 54 grms. was removed. The stone was covered with sharp spiculæ which must have caused a great deal of pain and easily accounted for the bleeding. The weight of the stone and irritation at the neck of the bladder evidently gave rise to the priapism. Patient has not been troubled with this condition since operation.

CASE 6.—C. J.—negro, Pullman porter, age 42, first seen March 28, 1905. He applied at Emergency Hospital for relief from retention of urine; gave history of having stricture dilated at various times during previous three years. He complained of frequent and painful urination and had passed blood occasionally but did not remember whether this followed passing of instruments.

Examination showed impassable stricture of the deep urethra. Perineal section was performed and stricture cut. Exploration of the bladder showed three small stones (No. 14). Patient made uneventful recovery and was discharged from the hospital in a week. I saw him ten months subsequent to the operation and, sounds having been passed at intervals during that period, he was in good condition and had nothing of which to complain. The question arising in this case aside from the retention, is how much of the history can be attributed to the stricture and how much to the stones?

CASE 7.—M. C.—Pole, age 8 years. Parents had noticed since child was three years old that he cried upon urination. As the symptoms became worse he was taken from one physician to another, each of whom gave medicine but without result. His parents moved to Chicago when the child was six years old, and he was in one of the hospitals there for four months being treated for cystitis. He was removed from the hospital and another physician consulted who advised a circumcision to alleviate his condition, which was done.

I saw the child July 10, 1905. He was weak, emaciated, high strung, lying in bed because he complained of great pain when he tried to walk. He could not walk erect but bent over in a crouching position. There was mucopurulent discharge from the urethra. I was unable to make a satisfactory examination at the time owing to the timidity of the child. I advised his removal to a hospital so that an *x*-ray picture might be procured, but to this the parents would not assent. I did not see the case again until September 24, 1905, at which time the parents were willing to have an examination under anesthetic. The child being anesthetised, the urethra and bladder irrigated, a Thompson searcher was passed and a diagnosis made of a large stone.

The bladder was opened suprapubicly and the bladder walls found much thickened. The stone (No. 2) weighing 16 grms. was removed complete with some difficulty. Because of some infection caused by the cystitis the opening did not heal readily. The patient was in bed for five weeks but otherwise made good recovery and since the operation has had none of his former pain.

CASE 8.—J. S.—German, age 62. First seen February 4, 1906. Patient was weak and emaciated; had been under treatment for Bright's disease. Gave history of having to pass urine frequently both day and night; urine foul, mucopurulent, albumin present, prostate enlarged. Catheterisation showed five ounces of residual urine. Urethra and vesical neck very sensitive and an examination with searcher was unsatisfactory owing to pain caused in using the instrument. His bladder was washed daily for a week with boric acid solution but without improvement and I feared his general condition was growing worse. I advised an operation on his prostate, and believing that he could not stand its removal in one sitting decided to open the bladder suprapubicly under cocaine and after drainage and irrigation get him in condition for the second operation. On February 12, his bladder was distended with boric acid solution and opened under cocaine. The walls were found much thickened and the mucus membrane congested. After washing out the bladder two small stones (Nos. 8 and 12) were discovered in the post prostatic pouch. The prostatic bar had been so sensitive that I had been unable to depress the searcher sufficiently to reach them. These were removed and the bladder drained by Dawbarn's method. It was washed out every four hours with a solution of alphosone, one to 1000. The patient suffered somewhat from shock but after five days the urine having cleared up considerably, a self retaining catheter was placed in the urethra and suprapubic drainage removed, the patient assuming a semi-reclining position. The bladder was now irrigated three times a day for another week and then twice daily. The wound did not entirely close for eight weeks. He had gained in strength, urine was clear and free from mucus; he catheterised himself every four hours and had no pain. During September his nephritis became worse and he died.

The interesting aspect of this case I think arises from the fact that even with his enlarged prostate he managed to get along fairly comfortably until the formation of the calculi, then the irritation so increased the cystitis that he began to lose ground. As soon as they were removed he picked up again, and had his kidneys been in better condition might have lived for some time.

CASE 9.—T. K.—American, age 47, consulted me July 13, 1906; gave history of having had urethral discharge and foul urine for three years; his bladder had been washed with boric acid solution by his physician and he had continued the washing which he found gave some relief but it was necessary to continue the same daily. Another physician diagnosticated enlarged prostate and advised rectal irrigation and massage.

Examination showed somewhat enlarged prostate. The urine was mucopurulent and upon microscopic examination showed number of blood cells with pus. A Thompson searcher detected small stone. A perineal section was made July 21, 1906, and a stone (No. 11) washed out. Upon inserting my finger to explore the bladder I found another (No. 10) down behind the prostate. This was sacculated and removed with some difficulty. This stone could not have been reached with a searcher or seen with an ordinary cystoscope. A retrograde cystoscope is the only instrument of which I know that could have shown it. The patient was out of bed on the fourth day and had his bladder washed daily for three weeks at which time the urine being clear treatment was discontinued. I saw the patient about three months afterwards and he reported no further trouble.

Case 10.—The following case is reported through the courtesy of Dr. Francis Carr. I saw the case with Dr. Carr at time of operation. F. S.—German, age 6; was first seen by Dr. Carr at his office on September 2, 1904. The mother gave a history of "doctoring and doctoring for more than three years." The trouble had been diagnosed as spinal trouble. The boy's abdomen had been swollen; he had fever and pain constantly, could walk but occasionally, wet his pants and the bed.

Physical examination showed that the boy could not stand up except with great difficulty, assuming a peculiar lateral lordosis; his abdomen was greatly distended; he could not lie on his back but turned over on his side, the spine still continuing in position of lordosis. On palpatating the bladder it was found to extend two inches above umbilicus and there was continual dripping of urine. Dr. Carr attempted to pass a catheter but the child screamed and became so excited that he was sent to the Emergency Hospital where he was anesthetised the following day, a searcher passed and a large stone diagnosed. On September 4th, a suprapubic cystotomy was performed and a stone (No. 4) weighing 16 grms. was removed. The child made uninterrupted recovery.

It is claimed that the Roentgen-ray examinations give less than three per cent. of error with practically no danger. The cystoscope is the most accurate in trained hands, and for the use of a great majority of the profession who do not possess either of these instruments and are not trained in their use, there still remains an instrument, the Thompson searcher, which is very accurate in a large percentage of cases if properly used. Its cost is within the reach of all and its use may clear up an otherwise puzzling case.

In closing I wish to emphasise the advisability of full exploration in all cases of chronic irritation of the bladder. These explorations may detect the presence of a calculus which has never been suspected. No harm can result from the use of the $x$-ray or the careful exploration of the bladder with a clean instrument.

403 FRANKLIN STREET.

## Figures and Tables

**Figure f1:**